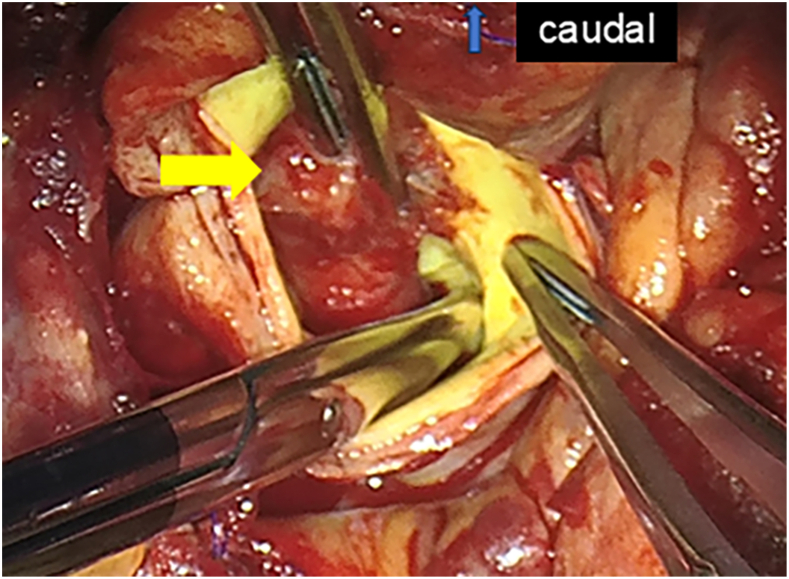# Floating Cast in the Ascending Aorta After Impella Removal

**DOI:** 10.1016/j.atssr.2025.11.001

**Published:** 2025-11-20

**Authors:** Takashi Tsuji, Kyokun Uehara, Taku Shirakami, Makoto Takehara, Hiroyuki Hara, Mamoru Hamuro

**Affiliations:** Department of Cardiovascular Surgery, Tenri Hospital, Nara, Japan

Thrombotic complications with Impella (Abiomed) are uncommon, occurring in about 0.7%.^1^ However, thrombus may occasionally mold itself around indwelling devices, leaving behind a hollow “cast” after removal.

A 71-year-old woman presented with acute myocardial infarction complicated by ventricular septal defect. Impella 5.5 support was initiated, which stabilized her circulation and allowed gradual recovery from severe heart failure. During Impella support, systemic anticoagulation was maintained with intravenous administration of heparin to achieve an activated clotting time of approximately 180 seconds, and a bicarbonate-based purge solution was used. Two weeks later, she underwent elective surgical closure of the ventricular septal defect under cardiopulmonary bypass, and Impella support was continued postoperatively.

**Figure 1:**
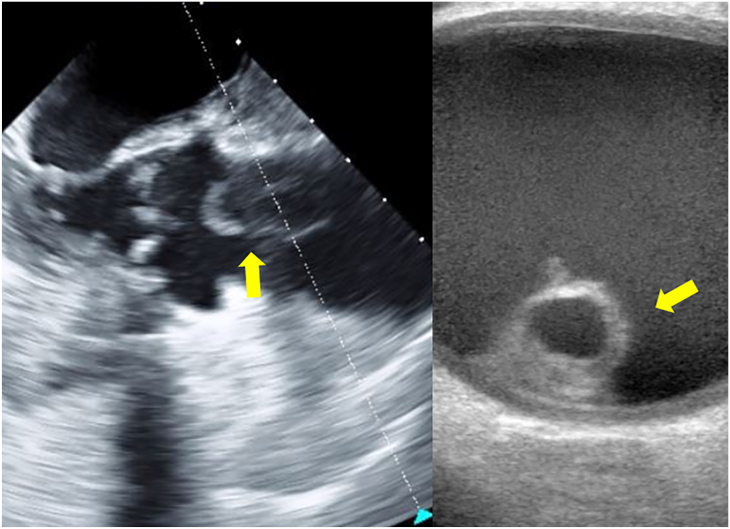

On postoperative day 3, after Impella removal, transesophageal echocardiography unexpectedly revealed a mobile mass floating in the ascending aorta (Figure 1, arrow). Given the imminent risk of embolization, emergent reoperation was performed. After repeated sternotomy and cross-clamping, a friable mass measuring 15 × 25 mm was found adherent to the posterior wall of the ascending aorta (Figure 2, arrow; Video). The lesion represented a fibrinous cast that had enveloped the Impella motor housing, remaining behind as a thrombotic shell after device withdrawal. The mass was shaved off and excised. Histopathologic evaluation confirmed fibrin-predominant thrombus. The patient recovered uneventfully.

**Figure 2:**